# The relationship between suicide and Islam: a cross-national study

**DOI:** 10.5249/jivr.v2i2.60

**Published:** 2010-06

**Authors:** Ajit Shah, Mahmood Chandia

**Affiliations:** ^*a*^University of Central Lancashire, Preston, United Kingdom and Consultant Psychiatrist, West London Mental Health NHS Trust, London, United Kingdom.; ^*b*^International School for Communities, Rights and Inclusion, University of Central Lancashire, Preston, United Kingdom.

## Abstract

**Background:**

Traditionally, suicide rates were low in Islamic countries. However, the process of modernization can erode the ties of the individual to society and lead to questioning of religion and weakening of faith. Therefore, a cross-national study with the null hypothesis that there will be no relationship between general population suicide rates and the percentage of adherents of Islam was undertaken.

**Methods:**

The correlation between general population suicide rates and the percentage of people in the general population adherent to Islam, whilst controlling for socioeconomic status and income inequality, was examined using cross-national data from the World Health Organization and the United Nations.

**Results:**

There were significant negative correlations between general population suicide rate and the percentage of people adherent to Islam in males and females whilst controlling for socioeconomic status and income inequality.

**Conclusions:**

Caution should be exercised in attributing a causal relationship and the direction of causality from this ecological study due to ecological fallacy. However, there is case to study further the impact of Islam on suicide by in-depth study of adherents Islam with and without suicidal ideation and behaviors.

## Introduction

There are important religious sanctions against suicide in Islam and suicide is illegal in several Islamic countries.^1,2^ Moreover, suicide has traditionally been poorly studied in Islamic countries.^1,3^ Furthermore, many Islamic countries do not collect national suicide statistics and/or they do not report such statistics to the World Health Organization (WHO).^1,4,5^ Suicide rates were low in migrants to England and Wales who were adherents of Islam.^6^ Overall, general population suicide rates have been reported to be low in individual Islamic countries.^1,2,7^ However, they were reported to be higher in European Islamic countries than South Asia and Middle Eastern Islamic countries;^4^ moreover, the range of suicide rates in Islamic countries was very wide and in three Islamic countries it was higher than in the United Kingdom.^4^ Elderly suicide rates were among the lowest only in the five Middle Eastern Islamic countries in a cross-national study of 87 countries.^5^

Modernization, a social and economic process consisting of three inter-related processes of industrialization, urbanization and secularization, influences general population suicide rates.^8^ Modernization, through these three processes, can erode the ties of the individual to society and lead to questioning of religion and weakening of faith.^8^ One unique cross-national study of 71 countries reported a negative correlation between general population suicide rates and the percentage of the population that was adherent to Islam whilst controlling for socio-economic factors.^9^ However, this study utilized data from 1970 and the data on suicide rates was ascertained from several diverse sources. It is possible that with modernization, this previously reported association between general population suicide rates and the percentage of the population adherent to Islam, using data almost four decades old, may currently be different. Therefore, a cross-national study with the null hypothesis that there will be no relationship between general population suicide rates and the percentage of adherents of Islam was undertaken using the latest available data. This study will deal with adherents of Islam in both Islamic and non-Islamic countries rather than only in Islamic countries. The reasons for this include: Islamic countries are difficult to accurately define (for example India has a substantial Moslem population, but is not acknowledged as an Islamic country); and focusing on Islamic countries would not allow the relationship between suicide rates and percentage of population who were adherents of Islam to be examined because there would be little variation in figures for the percentage of adherents of Islam across Islamic countries.

## Methods

Data on general population suicide rates for both sexes was ascertained from the WHO  website (www.who.int/whosis /mort/table1.cfm) . Data on suicide rates was collected for the latest available year and the median (range) of this latest year across the different countries was 2000 (1991-2002).

Data on the percentage of people in the general population adherent to Islam as their religion was ascertained from the United Nations Special Demography Yearbook (http://unstats.un.org/unsd/demographic/products/dyb/dybcensus/V2_table6.pdf) . This source does not provide the definition of adherents of Islam. However, it provides figures that were reported by individual countries and refer to those who are reported to follow the faith of Islam. These figures make no assumptions about the degree to which these followers follow Islam. The median (range) year for this data across the different countries was 2000 (1990-2004).

Data on per capita gross national domestic product (GDP) was ascertained from the WHO website (www.who.int/ countries/en/)  for the year 2002. The United Nations Development Programme website (http://hdr.undp.org/en/ media/HDR_20072008_Tables.pdf)  provided data on a measure of income inequality called the Gini coefficient. The median (range) for the latest year for this data was 2000 (1992 to 2004).

The univariate relationship between general population suicide rates and the percentage of people in the general population adherent to Islam was initially examined using Pearson’s correlation coefficient. Pearson’s correlation coefficient was chosen as the data on suicide rate was normally distributed. Normal distribution for general population suicide rates in both sexes was examined by using quantile-quantile (Q-Q) plot. This is a scatter plot with the quantiles of the scores on the x-axis and the expected normal scores on the y-axis. These plots for general population suicide rates were a near straight line suggesting normal distribution.

Socioeconomic status and income inequality have been reported to moderate the effect of religion on suicide rates in cross-national studies.^2,9,10^ Therefore, the independent relationship between general population suicide rates and the percentage of people in the general population adherent to Islam was further examined, by controlling for socio-economic status measured by GDP and income inequality measured by the Gini coefficient, using partial correlations.

## Results

Full data set for general population suicide rates and the proportion of people in the general population adherent to Islam was available for 27 countries.Table 1  illustrates the 27 countries studied along with data on male and female general population suicide rates, population size, GDP, Gini coefficient and percentage of adherents of Islam in the general population. The median (range) general population suicide rates in males and females were 19.8 (0-47.7) and 4.7 (0-17.9) per 100,000 population, respectively. There were significant negative correlations, using bivariate, analyses between general population suicide rate and the percentage of people adherent to Islam in males (r=-0.45, P=0.018) and females (r=-0.45, P=0.019).

**Table:** Table 1: **Some of the characteristics of the studied countries**

Country	Adherents of Islam(%)	Suicide rate Males	Suicide Rate Females	Population size GDP	Gini	coefficient
*Australia	1.1	20.1	5.3	1973100	28277	35.2
*Austria	4.2	30.5	8.7	8116000	28843	30.0
Bahrain	81.8	7.2	0.3	72400	18167	
Belize	0/1	0	0	256000	5748	
*Bosnia	42.8	20.3	3.3	4161000	3504	26.2
*Bulgaria	12.4	25.6	8.3	7897000	6738	31.9
*Canada	1.9	18.4	5.2	31510000	30429	33.1
*Croatia	1.3	30.2	10.0	4428000	8636	29.0
*Eygpt	94.1	0.1	0	71931000	3891	34.4
*Estonia	0.1	47.7	9.8	1323000	11836	37.2
*Finland	0.02	32.3	10.2	5207000	26614	26.9
*Ireland	0.5	21.4	4.1	3956000	32570	35.9
Mauritius	8.7	18.8	5.2	1221000	11046	
*New Zealand	0.4	19.8	4.2	3875000	31943	36.2
*Paraguay	0.02	3.9	1.7	5878000	4061	57.8
*Philipines	5.0	1.8	0.6	79999000	5231	34.1
*Portugal	0.1	18.9	4.9	10061000	18376	38.5
Qatar	7	7.5	0	0	610000	28467
*Romania	0.3	23.9	4.7	22334000	7468	30.3
St Kitts	0.1	0	0	42000	12175	
*Singapore	14.9	11.5	6.9	4253000	25588	42.5
*Slovenia	2.4	44.4	10.5	1984000	18687	28.4
Sri Lanka	0.9	43	17.9	19065000	3540	
*Switzerland	4.3	27.8	10.8	7169000	30723	33.1
*Thailand	4.6	13.5	3.7	62833000	7248	43.2
*Macedonia	33.3	10.3	4.5	2056000	5050	28.2
*Trinidad	5.8	22.2	4.7	1303000	11649	40.3

* Denotes countries involved in the second order (partial) correlations. Suicide rates are per 100,000 population in the relevent sex band

For analysis with partial correlations, full data set for general population suicide rates, the percentage of people in the general population adherent to Islam, GDP and the Gini coefficient was available only for 21 countries. There was a significant negative correlation between general population suicide rate and the percentage of people adherent to Islam in males (r=-0.53, P=0.019) and females (r=-0.46, P=0.046) whilst controlling for GDP and the Gini coefficient.

## Discussion

Some methodological issues need consideration. Cross-national data on suicide rates should be viewed cautiously because: data are not available from all countries;^1,11,12^ the validity of this data is unclear;^12,13^ the legal criteria for the proof of suicide vary between countries;^12,14^ some countries have poor death registration facilities;^14^ cultural and religious factors and stigma attached to suicide may lead to under-reporting of suicides,^12,15^ particularly in Islamic countries,^1,2,4^ where they may be reported as accidental deaths;^4^ and utilizing national aggregate data is likely to miss regional variations in suicide rates in individual countries.^16^ However, the best and latest available data from the WHO and the UNDP were used. The percentage of adherents of Islam across different countries does not provide any hard data on the degree to which these followers follow Islam.          

The significant negative correlations between general population suicide rate and the percentage of people adherent to Islam in males and females whilst controlling for socioeconomic status and income inequality rejected the null hypothesis. Although, these findings may be spurious due to the methodological issues described above, they were consistent with similar findings in a study almost four decades old,^9^ and this suggests that modernization has not weakened this relationship. Moreover, the current findings were also consistent with previous reports of low general population,^1,2,4,7^  and elderly^5^ suicide rates in individual Islamic countries. Therefore, potential explanations for the observed correlations are considered below.

The impact of religion on suicide rates have been explained by three hypotheses: Durkheim’s integration hypothesis; ^17^ the religious commitment hypothesis;^18,19^ and the network hypothesis. The low rate of suicide in Islam countries may be explained by the concept of life and views on suicide within Islamic teachings. Religious commitment in Islam and adherence to the normative structures of collectivism including collective goals, non-egoistic behavior, familial society and cohesive communities may be important in this context. These issues have been considered to be important in the evaluation of low suicide rates.^21^

The theme of suicide is conventionally expressed via two distinct phraseologies in classical Islamic literature located either within the sacred texts or within legal thought manuals. The two phraseologies are: qatl-al-nafs (lit. self-murder) and intihār (lit. cutting of the throat) (Quran, 4:29).^22,23^ The Quran, in the main, is free of explicit thematic reference to suicide. Consequently exegetical commentaries also contain little exhortation on the subject. However, the Quran does contain verses where the construct term ‘qatl-nafs’ appears. In such verses, in both classical and modern understandings, the context of the verses determines that they do not refer to the act of suicide.^22,24^ The theme of suicide is barely referred to within legal manuals. Its discussion is either restricted to extractive rulings on funeral prayers or peripherally mentioned under inheritance and marriage.^25^

The Quran emphasizes the sanctity of life and provides instructive guidance on the value of life and fulfilling the human role in this world whilst maintaining stability, patience and steadfastness in all circumstances (Quran, 17:33).^24^ Moreover, the collections of Prophetic traditions explicitly and in unequivocal terms not only proscribe suicide but condemn the perpetrator of such an act to eternal retribution in the form of incessant repetitions of the act and the anguish of the mode of suicide. The Prophetic traditions not only prohibit suicide but also explicitly deter from wishing for death.^26^

In light of the above, the low rate of suicide in Islamic communities may best be explained by an understanding of the concept of life in Islam within the matrix of the epistemology of Islamic Thought (i.e. Quran, Prophetic Traditions and Legal thought) because the concept of suicide is closely intertwined with the concept of life in Islam. Islamic Thought establishes that it is the Creator who has absolute power over human life and concerns. Violating this understanding entails not only committing a sin but also eternal damnation. Further, Islamic thought also advocates reward for those encouraging a good act and discouraging a reprehensible act (Q: 32:2; 32:5; 99:8). This is viewed as part of living a pious life. The collective understanding from scripture and legal thought asserts that the idea of suicide in Islam is extremely condemnable whilst preservation of life is virtuously commendable. However, it should be stated that the above interpretations are likely to be influenced by sectarianism and may be different for different branches of Islam.

This study reports a negative relationship between suicide rates and adherents of Islam across several countries. However, adherents of Islam do commit suicide and clinicians should carefully assess the risk of suicide in all patients including adherents of Islam.

Caution should be exercised in attributing a causal relationship and the direction of causality from the findings of this cross-sectional ecological study due to ecological fallacy. Another possibility is that there may be complex interaction between cultural, sociological and genetic factors.^27^ A study of suicide rates in major European countries between 1874 to 2000 reported variation in suicide rates within the countries over time, but the relative rates between countries were consistent.^27^ This suggested the possibility of interactions between genetic and other environmental factors and this requires further examination. However, there is case to study further the impact of Islam on suicide by in-depth study of adherents of Islam with and without suicidal ideation and behaviors.
